# Bioprocess Intensification Using Perfusion Culture for Recombinant Protein Production from *Lactococcus lactis*

**DOI:** 10.3390/microorganisms14102191

**Published:** 2026-10-01

**Authors:** Prashant Mainali, Jin Hao Tan, Pooi Leng Ho, Melvin Chua, Jiaxin Chua, Dave Siak-Wei Ow

**Affiliations:** 1 Bioprocessing Technology Institute (BTI), Agency for Science, Technology and Research (A*STAR), 20 Biopolis Way, #06-01 Centros, Singapore 138668, Singapore; prashant_mainali@a-star.edu.sg (P.M.); tan_jin_hao@a-star.edu.sg (J.H.T.); ho_pooi_leng@a-star.edu.sg (P.L.H.); melvin_chua@a-star.edu.sg (M.C.); 2 Department of Food Science & Technology, National University of Singapore, 2 Science Drive 2, Block S14, #06, Singapore 117543, Singapore; jiaxinchua85@gmail.com

**Keywords:** continuous biomanufacturing, basic fibroblast growth factor (FGF2), high-cell-density culture, *Lactococcus lactis*, perfusion, pareto front

## Abstract

Secretion of recombinant proteins from microbial cell factories is a promising strategy for recombinant protein biomanufacturing as it simplifies downstream processing. In this study, we sought to intensify the *Lactococcus lactis* high-cell-density culture, using basic fibroblast growth factor 2 (FGF2) as a model recombinant protein. We implemented a perfusion strategy using tangential flow filtration for cell retention, allowing the continuous removal of inhibitory metabolites while replenishing fresh nutrients. When conventional 2 × GM17 medium was used, the approach outperformed batch cultivation, achieving a 4.7-fold increase in biomass and a 3-fold increase in secreted FGF2, reaching a titer of 10,416 ± 892 µg·L^−1^. Concurrently, we developed a bioprocess model for *L. lactis* grown in a fortified spent cell culture medium, enabling systematic exploration of operating conditions. A Pareto front was generated for FGF2 titer against media usage, and perfusion profiles balancing both competing objectives were identified. An experimentally selected operating point validated the model predictions, yielding a final OD_600_ of 49.2 ± 0.3 and FGF2 titer of 2166 ± 161 µg·L^−1^ FGF2, which were 7.9-fold and 5-fold higher than batch process respectively. Overall, this work demonstrates a perfusion-based intensification strategy for *L. lactis* and highlights the utility of model-guided process decision-making to enhance productivity while reducing waste.

## 1. Introduction

Recombinant protein production in microbial systems offers advantages in scalability, rapid cultivation, and cost; however, downstream processing remains a challenge due to the difficulties of removal of host-protein contaminants. Protein secretion is promising as it eliminates the need for cell lysis, which simplifies purification workflows. Because only properly folded proteins transit the secretory pathway, secretion leads to functional products, minimizes inclusion body formation, and improves downstream recovery. Beyond the clear downstream benefits, secretion enables the integration of upstream and downstream operations, facilitating continuous product removal and process integration [1,2,3]. This compatibility with continuous biomanufacturing is increasingly important for improving productivity, consistency, and reducing manufacturing footprint, positioning protein secretion as a key strategy in next-generation microbial bioprocesses.

*Lactococcus lactis*, a Gram-positive bacterium with Generally Recognized As Safe (GRAS) status, is increasingly recognized as an attractive host for recombinant protein secretion [4]. It possesses a Sec-dependent secretion pathway, lacks endotoxins, and produces low levels of extracellular proteases, thereby minimizing the proteolytic degradation of secreted products [5]. In contrast, although *Escherichia coli* remains a dominant workhorse for recombinant protein production due to its rapid growth, genetic tractability, and high expression capacity, recombinant proteins are predominantly retained intracellularly. Efficient secretion in *E. coli* typically requires extensive strain and pathway engineering, and such engineered strains are often under significant metabolic stress, limiting their robustness in industrial processes [6]. Intracellular retention further exposes recombinant proteins to proteolytic degradation and promotes inclusion body formation [7], complicating downstream processing. *E. coli* can be cultivated to high cell densities in fed-batch processes [7] because carbon flux can be controlled through glucose-limited feeding. Although excess glucose leads to acetate formation via overflow metabolism, arising from limited respiratory capacity and proteome allocation constraints [8], this effect is not intrinsic and can be mitigated by maintaining low residual glucose concentrations. Careful feed-rate control aligns glycolytic flux with respiratory capacity, suppresses acetate accumulation, and enables process intensification, resulting in high biomass and high recombinant protein titers in fed-batch operations. While *L. lactis* offers clear advantages for protein secretion, its obligately fermentative metabolism and lactate production imposes a fundamental limitation in reaching high cell density [9]. Unlike Crabtree-positive organisms and organisms with overflow metabolism, *L. lactis* directs carbon to lactate even under low glucose and aerobic conditions due to the absence of a functional respiratory chain unless supplemented with heme and/or menaquinone, which completes its otherwise incomplete respiratory chain and can reduce lactate accumulation [10,11,12]. However, these supplements introduce additional media costs and may reduce the economic attractiveness of the process. Consequently, *L. lactis* typically reaches only modest biomass levels (OD_600_ ≈ 5–8) in batch cultures in the absence of heme [13,14,15], and this is hard to improve via feeding strategy unless feed is optimized for amino acids [16,17]. Although *L. lactis* enables extracellular protein secretion, product titers are low, and the inability to achieve high cell densities further constrains volumetric productivity and, ultimately, process economic viability. This necessitates bioprocess intensification strategies like a perfusion process to improve recombinant protein titer, hereby improving process economics and overall profitability [18].

Perfusion processes are widely practiced in mammalian cell culture [19], where the accumulation of inhibitory metabolites such as lactate necessitates continuous medium exchange to sustain growth and productivity. In contrast, microbial biomanufacturing is dominated by *E. coli* and *Saccharomyces cerevisiae*, for which high-cell-density fed-batch strategies are well established, limiting the adoption of perfusion-based strategies in microbial systems. Extending perfusion to microbial cultures also presents distinct engineering challenges. The substantially higher biomass concentrations achievable in microbial cultures increase solids loading and culture viscosity, which can accelerate membrane fouling, increase transmembrane pressure, and alter product transmission or sieving behavior during prolonged operation [20,21]. Nevertheless, recent advances demonstrate that these challenges do not preclude successful microbial perfusion. For example, prolonged recombinant protein production has been demonstrated in *Komagataella phaffii* using membrane- and acoustic-based cell-retention systems [22]. This study demonstrates that membrane- and acoustic-based cell retention can support extended microbial cultivation, despite the higher solids loading associated with microbial cultures.

*Lactococcus lactis* is an attractive microbial cell factory for heterologous protein production, particularly when secretion of the recombinant product into the extracellular medium is desired [23]. Its suitability as a production host is supported by well-characterized genetic tools and tightly regulated expression platforms, most notably the nisin-controlled gene expression (NICE) system [24]. Moreover, its Gram-positive cell-envelope architecture enables the direct secretion of recombinant proteins into the extracellular medium, potentially simplifying downstream recovery. Accordingly, considerable effort has been devoted to improving the protein secretion capacity of *L. lactis* through strategies such as the optimization of signal peptides and expression cassettes, engineering of the Sec-dependent secretion machinery and associated membrane components, and reduction in extracellular proteolysis [25,26,27]. Despite these advances in strain and secretion-pathway engineering, comparatively less attention has been given to intensifying the cultivation process itself as a means of increasing recombinant protein titers. In the context of *L. lactis*, however, perfusion presents an opportunity for improving process titer. By continuously removing growth-inhibitory lactate while retaining cells [28], perfusion cultivation can enable higher biomass densities than achievable in batch processes. We hypothesize that this increase in biomass will also translate into higher recombinant protein titers, as recombinant protein production is largely growth-coupled in *L. lactis*, while simultaneously preserving the intrinsic advantage of extracellular protein secretion. Central to perfusion operation is the cell-retention device, which enables continuous medium exchange while preventing biomass washout. Conventional retention technologies such as alternating tangential flow (ATF), tangential flow filtration (TFF), spin filters and acoustic separators have enabled high-cell-density cultures across numerous bioprocesses. However, these systems are often produced by few specialized suppliers produced in low volume, thus resulting in high cost. Hemodialysis cartridges represent an attractive alternative because they also have hollow-fiber membrane modules and are manufactured at industrial scale for decades for clinical blood purification [29]. These cartridges are produced under stringent quality standards with well-defined membrane properties, high manufacturing consistency, and global commercial availability. Their high-volume production has substantially reduced manufacturing costs relative to specialized bioprocess filtration devices while having robust membrane performance. Repurposing commercially available hemodialysis cartridges as cell-retention devices therefore offers a potentially economical strategy for microbial perfusion cultivation.

Mathematical modeling provides a complementary framework for the rational development of intensified microbial processes by quantitatively describing the dynamic relationships among biomass growth, substrate consumption, metabolite formation and recombinant protein production. Unstructured kinetic models are particularly useful for bioprocess applications because experimentally identifiable relationships, such as substrate-dependent growth, product or metabolite inhibition, and Luedeking–Piret-type product formation, can be incorporated without requiring detailed knowledge of intracellular metabolism [30]. Such frameworks can be extended to recombinant systems by incorporating product-formation kinetics and changes in cellular production capacity, enabling model-based prediction and process control [31]. Accordingly, kinetic models have been widely used to guide experimental design, support process optimization, and generate mechanistic insight [32]. Across upstream operations, models have been applied to optimize feeding strategies in *E. coli* [33], guide perfusion process development [34], and evaluate how dilution rate influences chemostat process parameters [35]. More detailed metabolic models, including dynamic flux-balance approaches, have similarly been applied to identify metabolic bottlenecks and guide the process intensification of recombinant microbial cultures [36]. Once experimentally parameterized, these models provide an in silico framework for evaluating operating strategies that would otherwise be costly or time-consuming to investigate experimentally.

Despite this, modeling studies focused specifically on microbial perfusion remain relatively underexplored, given the limited use of perfusion process in microbial biomanufacturing. At the same time, high media usage in perfusion processes can contribute to operational costs. On this note, perfusion bioprocess development frequently involves balancing multiple, often conflicting, performance criteria. Maximizing biomass concentration may not maximize recombinant protein production, while increasing productivity can be accompanied by higher substrate wastage, increasing operating costs. Consequently, optimization based on a single response often identifies solutions that are suboptimal when overall process performance is considered. Conventional single-objective optimization may therefore be insufficient when improvements in one process metric, such as productivity, are accompanied by trade-offs in product titre, substrate utilization, process duration, or operational requirements. Multi-objective optimization addresses this challenge by simultaneously optimizing multiple competing objectives and identifying a set of non-dominated solutions, commonly referred to as the Pareto front. Rather than producing a single optimum, Pareto optimization provides a set of equally optimal operating conditions, in which improvement in one objective can only be achieved at the expense of another. This framework enables rational decision-making by allowing researchers to select operating conditions that best satisfy technical, economic or manufacturing priorities [37]. Multi-objective approaches have increasingly been applied to microbial fermentation to balance competing objectives such as productivity, yield, product concentration, and resource utilization [38], supported by advances in mechanistic modeling, evolutionary optimization algorithms, and machine-learning methods. In a recent work, Korang Yeboah et al. (2026) [39] developed a feasibility-aware dynamic Pareto optimization framework for consolidated bioprocessing of lignocellulosic biomass, simultaneously considering ethanol titre, volumetric productivity, substrate conversion, process duration, and operating severity. The resulting Pareto solutions revealed trade-offs among process performance and operational requirements and enabled identification of dynamic operating strategies that improved multiple performance metrics relative to criterion-specific static conditions [39]. In microbial perfusion, increasing the perfusion rate can improve recombinant protein production by supporting cell growth and limiting metabolite accumulation, but at the cost of greater media consumption. Multi-objective optimization provides a practical way to evaluate these competing effects. Integrating mechanistic models with Pareto optimization therefore can provide a framework for identifying operating conditions that balances competing objectives [40]. We therefore hypothesize that mechanistic modeling can be leveraged to rationally design microbial perfusion strategies that maximize recombinant protein titer while minimizing media waste, improving both economic and environmental sustainability of the perfusion process.

Accordingly, this study pursued two complementary objectives: (i) the application of perfusion cultivation using a repurposed hemodialysis cartridge as a cell-retention device to enhance recombinant protein production in *L. lactis*, and (ii) the integration of mechanistic modeling with Pareto-based multi-objective optimization to guide perfusion process operation and balance productivity with media wastage.

## 2. Materials and Methods

### 2.1. Bacterial Strains and Protein Expression Plasmid

The bacterial strain used in this study was *L. lactis* NZ9000 carrying pNZ9148 vector (Boca Scientific, Dedham, MA, USA). The FGF2-G3 gene [41] was codon-optimized for *L. lactis* and placed under the transcriptional control of the nisin-inducible promoter, P_nis_, within plasmid pNZ8148. For brevity, the FGF2-G3 is hereafter referred to as FGF2. To facilitate the extracellular secretion of FGF2, USP45 secretion signal and secretion propeptide pp1 tags [4] were incorporated upstream of the FGF2 coding sequence. In addition, six Histidine residues (6 × His) were included at the N-terminal for protein purification.

### 2.2. FGF2 Purification and Concentration

The cells were lysed using sonication to recover intracellular FGF2. Cell pellets were washed and subsequently lysed on ice bath using five sonication cycles, with each cycle consisting of 10 s of sonication followed by 10 s cooling on ice bath. The lysate was then centrifuged to remove insoluble cell debris, and the remaining soluble fraction was retained for further purification or Western blot analysis. For recovery of the secreted FGF2, 50 mL of cell-free culture medium after centrifugation was concentrated using 10 kDa molecular-weight-cutoff (MWCO) centrifugal protein concentrator (Merck Millipore, Billerica, MA, USA). The concentrated extracellular fraction was subsequently used for purification. Ni-NTA affinity chromatography (Qiagen, Hilden, Germany) was used for the purification of both intracellular and secreted FGF2. Initially, a column was packed with a 2 mL Ni-NTA slurry. Thereafter, intracellular soluble lysate or the concentrated extracellular fraction was loaded in the column and an equal amount of buffer (50 mM NaH_2_PO_4_, 300 mM NaCl, 10 mM Imidazole, pH 8) was added. Then, the column that had the Ni-NTA slurry, either intracellular soluble lysate or the concentrated extracellular fraction, and buffer was incubated at 4 °C for 1 h to facilitate binding. Following binding, 10 mL of wash buffer (50 mM NaH_2_PO_4_, 300 mM NaCl, 20 mM Imidazole, pH 8) was used to remove non-specifically bound proteins from the column. Bound FGF2 was subsequently recovered using an elution buffer (50 mM NaH_2_PO_4_, 300 mM NaCl, 500 mM Imidazole, pH 8). The resulting 8 mL eluate was concentrated to a final volume of 200 µL using a Millipore centrifugal protein concentrator (Merck Millipore, MA, USA). The protein concentration was determined using the Bradford assay, with Bovine Serum Albumin (BSA) as the calibration standard.

### 2.3. SDS-PAGE and Western Blot

For protein visualization, 13 µL of each sample was mixed with 7 µL of loading buffer, heat-treated, and separated on 4–12% NuPAGE^TM^ Bis-Tris Gels (Invitrogen, Carlsbad, CA, USA). For SDS-PAGE analysis, following electrophoretic separation, the gel was rinsed with Milli-Q water and stained using InstantBlue^TM^ Coomassie Protein Stain (Abcam, Cambridge, UK) to visualize the protein bands. For Western blot analysis, proteins separated by SDS-PAGE were transferred from the gel onto a nitrocellulose membrane using a semi-dry blotting system at 20 V for 20 min. The membrane was then probed with a Basic FGF Rabbit monoclonal antibody (Abcam, Cambridge, UK), followed by incubation with an anti-rabbit HRP linked IgG antibody (Abcam, Cambridge, UK). Protein bands were detected using Clarity Western ECL substrate (Biorad, Hercules, CA, USA), and chemiluminescent images were acquired using an ImageQuant^TM^ LAS 500 imaging system (General Electric, Fairfield, CT, USA).

### 2.4. Media

Experiments were conducted using either 2 × M17 medium (DIFCO^TM^, Sparks, MD, USA) supplemented with 2% (*w*/*v*) glucose, hereafter referred to as 2 × GM17 or fortified medium, prepared according to a previously reported formulation [42]. To prepare the fortified medium, spent medium was collected from suspension cultures of chicken fibroblast DF-1 cells (ATCC CRL-12203^TM^) grown in a chemically defined medium. Following 3–4 days of cultivation, the spent medium, containing unconsumed nutrients and accumulated metabolites, was harvested, and supplemented with a non-animal-origin formulation comprising yeast extract (<1.25% *w*/*v*), soy peptone (<0.3% (*w*/*v*)), and two amino acids (<80 mM in total), as described in a previous study [29]. Compared to the previous study, the formulation used in this work was further supplemented with 10 mM each of asparagine, histidine, and proline. Chloramphenicol was added into the medium at a final concentration of 10 µg/mL to maintain selection pressure. The composition of the fortified spent medium, prior to glucose adjustment, and conventional growth media (2 × GM17) was characterized using a BioProfile 100 Plus analyzer (Nova Biomedical, Waltham, MA, USA). Total protein concentration and free amino acids were quantified using Bradford and ninhydrin assays, respectively. A comparative summary of the measured medium components is provided in the Supplementary Section (SI, Table S1).

### 2.5. Batch Fermentation

Fermentation experiments were performed in a CloudReady 1.5 L parallel bioreactor system (TJXBIO, T&J Bioengineering, Shanghai, China), carried out at a working volume of 750 mL of a fortified formulation. Batch experiments in a fortified formulation were used to parameterize the model. The culture pH was maintained at 7 using a PID-based control, with additions of 14% NH_4_OH and 1 M H_2_SO_4_ as needed. Anaerobic conditions were established by sparging the bioreactor with nitrogen. Agitation and temperature were kept constant at 300 rpm and 35 °C, respectively, throughout the fermentation. The initial cell density (OD_600_) at the start of the experiment was 0.15. Once the culture reached an OD_600_ of approximately 0.4, recombinant protein expression was induced by adding nisin to a final concentration of 100 ng/mL. The time point following nisin addition was designated as 0 h.

### 2.6. Perfusion Process

Perfusion cultivations were conducted in the same stirred-tank bioreactor used for batch experiments, operated at 35 °C and 300 rpm. The working volume was 500 mL. The perfusion feed medium was either 2 × GM17 or the fortified formulation as described in the method section with nisin (100 ng/mL) and chloramphenicol (10 µg/mL). Initially, the process was started as a batch process with an initial OD of 0.25, and when the culture reached OD 0.5, nisin was added to a concentration of 100 ng/mL. The time point following nisin addition was designated as 0 h. A repurposed renal hemodialysis cartridge ELISIO^TM^ (Nipro, Selangor, Malaysia) was used as the cell-retention device (Figure 1). The cartridge consists of a polyethersulfone hollow-fiber membrane (surface area 2100 cm^2^, length 29 cm, internal fiber diameter 200 µm, membrane fiber wall 40 µm). The membranes are housed by the polypropylene shell. The ultrafiltration coefficient (KUF) is 25 mL/h/mmHg and with a maximum transmembrane pressure of 500 mmHg. Membrane permeability is characterized by the sieving coefficient (SC), defined as the ratio of the solute concentration in the filtrate to that in the feed or bulk solution under specified filtration conditions. Thus, an SC approaching 1 indicates that a solute readily passes through the membrane, whereas an SC approaching 0 indicates strong retention. For the ELISIO^TM^ membrane, the reported sieving coefficients are approximately 0.88 for vitamin B12 (~1.36 kDa), 0.44 for inulin (~5 kDa), <0.01 for myoglobin (~17 kDa), and <0.01 for albumin (~66.5 kDa). During perfusion, the culture broth was circulated through the cartridge while permeate was withdrawn, retaining cells and higher molecular weight solutes within the bioreactor. For all experiments, the culture recirculation rate through the cartridge was maintained at 50 mL min^−1^. Transmembrane pressure (TMP) was not directly monitored during these experiments. Permeate withdrawal was driven by negative pressure generated using a peristaltic pump, with the pump calibrated to achieve the desired permeate flow rate.

For perfusion runs in 2 × GM17 medium, perfusion was performed in repeated semi-continuous exchange cycles. Broth was circulated through the cartridge and permeate withdrawn until the working volume decreased to 100 mL, after which circulation was paused, and fresh medium was added to restore the working volume to 500 mL. The culture was then allowed to grow until the pH reached 5.6, and the exchange cycle was repeated for a total of five cycles. For perfusion runs in fortified medium, the culture was first operated in batch mode for 4.5 h, after which continuous perfusion was initiated and continued for another 8 h by feeding fresh medium and withdrawing permeate at equal flow rates to maintain a constant working volume. The flow/permeate rates used were obtained based on model simulation.

### 2.7. Kinetic Model

A mathematical model was developed to describe the growth and recombinant protein production of *L. lactis* cultivated in the fortified medium formulation [42]. The model comprises a system of Ordinary Differential Equations (ODEs), as presented in Equations (1), (3), (5), (7) and (9), describing the temporal changes in cells, glucose, lactate, intracellular FGF2, and secreted FGF2 concentrations for *L. lactis* grown in 35 °C. Model parameterization and numerical simulations were performed in MATLAB 25.2.0.2998904 (R2025b) (MathWorks Inc., Natick, MA, USA).

(1) $$\frac{d\;\left[X\right]}{dt}=\mu \times X$$

where

(2) $$µ=qS\times Y_{XS}\times \frac{1}{1+\left(\frac{L^{n1}}{L_{i,µ}^{n1}}\right)}$$

µ is specific growth rate;
qS is specific glucose consumption rate;
$Y_{\mathrm{XS}}$ is biomass yield coefficient from glucose;
$L_{i,µ}$ is lactic acid inhibition in growth rate;
n1 is hill coefficient.

(3) $$\frac{d\;\left[S\right]}{dt}=-qS\times Cells+D\times \left(S_{i.S}-S\right)$$

where

(4) $$qS=qS_{max}\times \frac{S}{K_{S}+S}\times \frac{1}{1+\left(\frac{L^{n2}}{L_{i,qS}^{n2}}\right)}$$

qS is specific glucose uptake rate;
$K_{s}$ is saturation constant for glucose;
${\mathrm{qS}}_{\max}$ is maximum specific glucose consumption rate;
$L_{i,\mathrm{qS}}$ is inhibition constant from lactate on glucose uptake;
n2 is hill coefficient;
$S_{i.S}$ is glucose concentration in feed;
D is the dilution rate.

(5) $$\frac{d\;\left[L\right]}{dt}=qL\times X-D\times L$$

where

(6) $$qL=qL_{max}\frac{qS}{K_{SL}+qS}$$

qL is specific lactate secretion rate;
${\mathrm{qL}}_{\max}\;$ is maximum specific lactate production rate;
$K_{\mathrm{SL}}$ is saturation constant.

(7) $$\frac{d\;\left[FGF2_{\mathrm{intra}}\right]}{dt}=qFGf2_{\mathrm{intra}}\times X-qFGF2_{\sec}\times X$$

where

(8) $$qFGf2_{intra}=\alpha_{i}\;µ\times \frac{1}{1+\left(\frac{L^{n3}}{L_{i,qP_{intra}}^{n3}}\right)}$$

$qFGF2_{intra}$ is specific intracellular FGF2 production rate,
$\alpha_{i}$ is growth-associated specific intracellular FGF2 production rate,
$L_{i,qP_{intra}}$ is lactate inhibition term for intracellular FGF2 production,
n3 is hill coefficient.

(9) $$\frac{d\;\left[FGF2_{sec}\right]}{dt}=qFGF2_{sec}\times X$$

where

(10) $$qFGF2_{sec}=\alpha_{s}µ\times \frac{FGF2_{i}}{K_{FGF2_{i}}+FGF2_{i}}\times \frac{1}{1+\left(\frac{L^{n3}}{L_{i,qFGF2_{sec}}^{n3}}\right)}\;\;\;\;\;\;\;\;\;\;\;\;\;\;\;\;\;\;\;\;\;\;\;\;\;\;\;\;\;\;\;\;\;\;\;\;+\beta_{s\;}\times \frac{FGF2_{i}}{K_{FGF2_{i}}+FGF2_{i}}\times \frac{1}{1+\left(\frac{L^{n3}}{L_{i,qFGF2_{sec}}^{n3}}\right)}$$

$qFGF2_{sec}$ is specific secreted FGF2 production rate,
$\alpha_{s}$ is growth-associated specific secreted FGF2 production rate,
$\beta_{s}$ is non-growth-associated specific secreted FGF2 production rate,
$L_{i,qFGF2_{sec}}$ is lactate inhibition term for secreted FGF2 production,
n3 is hill coefficient.

In the batch process, D equals 0 for Equations (3) and (5), while for the perfusion process, D is given by:

(11) $$D=\;\frac{F}{V}$$

where V, i.e., working volume, is constant, while F is perfusion feed and permeate rate, which are increased exponentially during cultivation according to:

(12) $$F\left(t\right)=\;F_{0}\exp\left[K_{f}\left(t-\;t_{0}\right)\right]$$

where $F_{0}$ is the initial perfusion feed/permeate rate, $K_{f}$ is the perfusion ramp coefficient, and $t_{0}$ is the onset time of perfusion.

### 2.8. Parameterization

The model contained 16 unknown parameters, which were estimated using a local optimization approach implemented in MATLAB R2025b (MathWorks Inc., MA, USA). Parameter estimation was carried out using fmincon, MATLAB’s built-in gradient-based solver for constrained nonlinear optimization. Initial estimates were specified for each parameter together with lower and upper bounds to define the parameter space. The optimization procedure selected the parameter set that minimized the weighted sum of squared residuals between the experimentally measured values and the corresponding model predictions, according to the objective function defined in Equation (13).

(13) $$f\left(p\right)=\underset{i,j}{\sum }w_{i}{\left(x_{i,j}-y_{i,j}\left(p\right)\right)}^{2}$$

where p denotes the parameter’s set to be estimated by the optimization algorithm, $x_{i,j}$ represents the experimentally measured concentration of species $x_{i}$ at time point j, and $y_{i,j}\left(p\right)$ is the corresponding model-predicted value for a given parameter set p. The normalization factor for $x_{i}$ was defined as the inverse of the maximum value of $x_{i}$, which is represented as $w_{i}$.

Experimental data obtained from three batch cultivations were used to estimate the 16 unknown model parameters. To account for experimental variability and uncertainty, 100 new datasets were generated by drawing 100 Monte Carlo (MC) samples from a normal distribution, N(x,σ), where *x* represents the mean of the measured experimental data at each time point, and *σ* is the corresponding standard deviation. The parameter estimation procedure was independently performed for each of the 100 generated datasets, resulting in 100 estimated parameter sets. For model simulations, parameter values were randomly sampled from the estimated distributions to generate 20 different parameter combinations. The 16 parameters of the model, their descriptions, units, and estimated parameter values, along with their initial guesses and respective lower and upper bounds applied during parameter optimization, are provided in the Supplementary Section (SI, Table S2).

### 2.9. Pareto Front

Multi-objective optimization was performed by evaluating candidate perfusion feed profiles and selecting non-dominated solutions based on competing objectives. A deterministic grid-search approach was used, in which the decision variables $F_{0}$ (initial perfusion feed rate) and $k_{f}$ (exponential feed constant) were sampled over 40 evenly spaced values, resulting in a total of 1600 candidate operating conditions. Specifically, $F_{0}$ was varied from 0 to 450 mL h^−1^, and $k_{f}$ from 0 to 0.045 h^−1^. Each $\left(F_{0},k_{f}\right)$ pair defined a unique perfusion trajectory according to $F\left(t\right)=F_{0}\exp\left(k_{f}t\right)$. For every candidate profile, the resulting bioprocess was simulated, exploring full design space for 8 h using the ODE-based dynamic model. All simulations were initialized using identical process conditions. The initial biomass, glucose, and lactate concentrations were OD 5, 2 g L^−1^, and 5 g L^−1^, respectively. The initial intracellular and extracellular FGF2 concentrations were set to zero. The initial glucose waste was initialized at zero. The reactor volume was fixed at 0.5 L and the perfusion feed substrate concentration was set to 10 g L^−1^. For the Pareto front analysis, only extracellular FGF2 produced during the simulated perfusion phase was considered; FGF2 produced during the preceding batch cultivation phase was excluded from the objective calculation.

Two objectives were evaluated: (i) maximization of the final secreted FGF2 titer, and (ii) minimization of glucose waste. Cumulative glucose waste was calculated as the total glucose mass discharged in the perfusion outlet over the cultivation period, according to $\frac{d\;\left[Waste\right]}{dt}$ = F(t)S(t), where F(t) is the perfusion flow rate (L h^−1^), and S(t) is the reactor glucose concentration (g L^−1^). Accordingly, total glucose waste was expressed in grams. A feasibility constraint was additionally imposed such that the total perfused medium volume over the cultivation period did not exceed (~3500 mL). Only feasible candidates satisfying V≤3500 mL were retained for Pareto analysis.

To apply a minimization-based Pareto routine, the objective vector was expressed as [ - *Final FGF2 titer (µg L^−^*^1^), *total glucose waste (g*)]. Minimizing the negative of *Final FGF2 titer (µg L^−^*^1^*)* is mathematically equivalent to maximizing the final FGF2 titer, whereas *total glucose waste (g*) was minimized directly. Pareto front selection was implemented using a non-dominated sorting procedure based on pairwise dominance checks: a candidate solution was classified as dominated if another feasible solution existed that was no worse in both objectives and strictly better in at least one objective [43]. The Pareto front was defined as the set of all non-dominated feasible solutions and represented the best achievable trade-offs between maximizing secreted FGF2 and minimizing glucose waste under the imposed feed-capacity constraint [44]. All the codes were implemented in MATLAB 25.2.0.2998904 (2025b) (MathWorks Inc., MA, USA).

## 3. Results

### 3.1. Kinetic Model

An unstructured kinetic model was developed to capture the batch cultivation of recombinant *L. lactis* to simultaneously capture cell growth, glucose consumption, lactate production, intracellular FGF2 accumulation, and extracellular FGF2 secretion. Model parameters were estimated using experimental data obtained from batch cultivations with three different starting glucose concentrations (6, 10, and 20 g L^−1^), allowing the model to capture cellular responses over a broad range of substrate availability. The model assumes that glucose is the primary growth-limiting substrate, while lactate is the dominant inhibitory metabolite affecting growth rate and recombinant protein production. Consequently, glucose uptake was described using Monod kinetics with lactate inhibition, allowing substrate availability and lactate accumulation to jointly regulate growth. Biomass growth was assumed to be directly coupled to glucose uptake, reflecting the fermentative metabolism of *L. lactis*, where glucose serves as the principal carbon and energy source. Lactate formation was linked to glucose uptake because homolactic fermentation is the predominant metabolic pathway under the cultivation conditions employed. Intracellular FGF2 synthesis was modeled as a growth-associated process, whereas secretion was represented using both growth-associated and non-growth-associated components to account for continued protein export after growth slowed. Both intracellular synthesis and secretion were assumed to be inhibited by lactate accumulation, consistent with the negative physiological effects of organic acid accumulation on recombinant protein production. However, the model does not account for membrane fouling, partial cell leakage, incomplete protein retention, maintenance metabolism, intracellular regulatory networks, or shifts in metabolic flux distribution, which we acknowledge to further improve the model.

Overall, the kinetic model accurately reproduced the experimental profiles for all measured state variables across the three cultivation conditions (Figure 2). Cell growth followed a characteristic sigmoidal pattern, with biomass increasing rapidly during the exponential phase before reaching a stationary phase. Increasing the initial glucose concentration increased the final biomass concentration from approximately OD_600_ 5.9 at 6 g L^−1^ glucose to approximately OD_600_ 6.6 and 7.6 at 10 and 20 g L^−1^ glucose, respectively. The model closely matched these trends. The predicted glucose consumption profiles also agreed well with the experimental observations. Cultures initiated with 6 and 10 g L^−1^ had depleted glucose within approximately 5–7 h. In contrast, glucose remained in excess throughout the 20 g L^−1^ cultivation, with a substantial residual concentration still present after 22 h. This indicates that, under the highest substrate condition, cessation of growth was not caused by carbon limitation but rather by other physiological constraints, such as accumulation of inhibitory metabolites. Consistent with the predominantly homolactic metabolism of *L. lactis*, lactate accumulated rapidly during exponential growth and reached higher final concentrations as the initial glucose concentration increased. The model reproduced lactate formation across all three conditions, supporting the assumption that lactate production is closely coupled with glucose metabolism and biomass formation. Intracellular FGF2 accumulated rapidly during the exponential growth phase and reached a plateau shortly after cultures entered stationary phase. The experimental results suggest that intracellular recombinant protein synthesis is primarily growth-associated, with most protein production occurring while cells are actively dividing. In contrast, secreted FGF2 continued to increase after intracellular FGF2 had largely plateaued. Although most extracellular protein accumulated during active growth, continued secretion after biomass formation slowed indicates the presence of both growth-associated and non-growth-associated contributions to product formation. Interestingly, increasing the initial glucose concentration beyond 10 g L^−1^ did not improve either intracellular or secreted FGF2 production. Collectively, these results demonstrate that the kinetic model captures the dynamic relationships among biomass growth, substrate consumption, metabolite production, intracellular protein accumulation, and extracellular protein secretion during the initial phase (approximately 0–8 h) across the glucose concentrations evaluated. However, deviations between the model predictions and experimental measurements became more apparent at extended cultivation times, particularly for cultures initiated with 20 g L^−1^ glucose.

### 3.2. Hemodialyzer Cartridge Is Repurposed as Cell-Retention Device for Microbial Perfusion Process

Perfusion culture requires an effective cell-retention device to maintain biomass within the bioreactor while continuously removing spent medium containing depleted nutrients and inhibitory metabolites. Conventional cell-retention strategies include hollow-fiber membrane [45], acoustic settlers [46], gravity-based settlers [47], centrifugation-based systems [48], and spin filters [49]. In this study, we investigated the feasibility of repurposing a commercially available hemodialyzer cartridge, originally designed for clinical renal dialysis, as a low-cost hollow-fiber cell-retention device for microbial perfusion. In hemodialysis, blood is circulated through semi-permeable hollow fibers, which retain blood cells and macromolecules while allowing low-molecular-weight waste solutes (e.g., urea and creatinine) to be removed across the membrane. Solute transport is primarily driven by diffusion along concentration gradients, with ultrafiltration additionally enabled through transmembrane pressure. This mechanism is conceptually analogous to perfusion, where the objective is to retain cells while enabling the continuous withdrawal of permeate containing extracellular metabolites and spent medium components. Given the generally higher shear tolerance of microbial cells compared to mammalian cells, we hypothesized that a hemodialyzer cartridge which has an internal diameter of 200 µm is still tolerable for microbial perfusion. Unlike clinical dialysis, which employs dialysate flow on the shell side to maintain diffusion gradients, our perfusion configuration withdraws permeate by applying controlled negative pressure on the shell side using a peristaltic pump, thereby enabling medium exchange while retaining microbial biomass.

The hemodialyzer cartridge was integrated into the perfusion setup as illustrated in Figure 1. Cultivations were performed using 2 × M17 supplemented with 20 g L^−1^ glucose (2 × GM17), a commonly used complex medium for *L. lactis* in laboratory. The process was initiated in batch mode. Upon reaching a pH of approximately 5.6, medium exchange was performed by withdrawing ~80% of the culture volume through the cartridge, followed by the addition of 400 mL of fresh medium. After replenishment, the culture was allowed to recover and continue growth. This cycle was repeated five times. The hemodialyzer cartridge functioned effectively as a hollow-fiber cell-retention device in the microbial perfusion setup (Figure 1). Across all sampling time points, the permeate exhibited negligible optical density (OD_600_ ≈ 0), indicating effective cell retention of *L. lactis* biomass (Figure 3a) and confirming the integrity of the membrane barrier under operating conditions. In addition to retaining cells, the cartridge also retained secreted FGF2 (Figure 3b), which is advantageous as it enables in situ concentration of the product within the retentate. Using this approach, the culture reached an optical density of 23.76 ± 1.57, representing a 4.7-fold increase compared with batch cultivation (5.03 ± 0.59) across three biological replicates (Figure 3c). This increase in OD concentration was statistically significant (unpaired two-tailed Student’s *t*-test, *p* = 3.71 × 10^−5^). Moreover, the final concentration of secreted FGF2 reached 10,416 ± 892 µg L^−1^, corresponding to an approximately 3-fold improvement over batch operation (3460 ± 1647 µg L^−1^; Figure 3d). This increase in FGF2 concentration was statistically significant (unpaired two-tailed Student’s *t*-test, *p* = 0.003011). As shown in Figure 3e, Western blot analysis of culture supernatants collected at different time points during perfusion revealed a progressive increase in secreted FGF2, with the highest signal observed at the final sampling point. This trend indicates the effective retention of FGF2 within the retentate and its gradual enrichment over time. Consistent with these results, the purified samples analyzed by SDS–PAGE at the final time point (Figure 3f) showed a visibly stronger FGF2 band in the perfusion condition compared to batch, both in the undiluted sample and after 10-fold dilution, confirming the substantially higher secreted titer achieved under perfusion. Collectively, these results demonstrate that the hemodialyzer cartridge can be repurposed as a robust and low-cost cell-retention device for microbial perfusion, enabling process intensification while simultaneously supporting the in situ enrichment of a secreted 17 kDa recombinant protein.

### 3.3. Pareto Front for Decision-Making During Perfusion Process

The higher volumetric productivity in perfusion culture can reduce production cost per unit mass of product of interest than a batch processes. However, continuous media exchange in the perfusion process generates a substantial volume of spent medium, which may still contain appreciable amounts of unconsumed nutrients. Analysis comparing perfusion and fed-batch processes has suggested that, although perfusion does have an economic benefit, its environmental footprint may be greater because of increased water demand and the extensive use of single-use materials [50]. Thus, it is necessary to reduce waste while also improving the protein yield.

In the perfusion process, this challenge represents a classic multi-objective trade-off. Lower perfusion rates reduce medium usage and nutrient loss but can limit productivity by increasing the accumulation of inhibitory metabolites and restricting nutrient availability. Conversely, higher perfusion rates improve performance and stabilize the culture environment but increase nutrient waste and spent medium volume. Because improvements in one objective can directly worsen the other, identifying a single “optimal” operating condition is not meaningful; instead, the goal is to identify operating policies that provide the best compromises. To address this, a bioprocess model was used to simulate the range of perfusion strategies, controlled by (i) the initial perfusion rate, and (ii) the rate at which perfusion rate increased over time. Two objectives were evaluated: maximizing secreted FGF2 titer and minimizing nutrient waste. In this analysis, residual glucose in the harvest stream was used as a proxy for overall nutrient wastage, the fact being that glucose is typically a dominant carbon and energy source and correlates with excess feeding and other unused medium components like amino acids. The simulation results generated a Pareto front representing the set of non-dominated operating strategies (Figure 4a). Solutions on this front define the boundary of best achievable trade-offs, where any further reduction in glucose wastage requires a decrease in secreted FGF2 titer, and any further increase in FGF2 secretion requires greater nutrient (glucose) loss. Importantly, the Pareto front exhibited a clear knee region, corresponding to operating conditions where substantial reductions in nutrient wastage could be achieved with only modest penalties in FGF2 secretion. Beyond this knee point, further improvements in one objective resulted in disproportionately large sacrifices in the other. Therefore, the knee point represents an engineering compromise and provides a rational basis for selecting perfusion policies that balance productivity with sustainability. Figure 4b,c presents heatmaps of the simulated final secreted FGF2 titer and glucose waste across the explored perfusion design space, where $F_{0}$ was varied from 0 to 450 mL h^−1^ and $k_{f}$ from 0 to 0.045 h^−1^. Each $\left(F_{0},k_{f}\right)$ pair defines a unique perfusion trajectory according to $F\left(t\right)=F_{0}\exp\left(k_{f}t\right)$. The blue arrow indicates the selected operating point ($F_{0}$ = 320 mL h^−1^ and $k_{f}$ = 0.035 h^−1^), which lies near the elbow region and provides a balanced trade-off between maximizing FGF2 production and minimizing glucose waste. This condition was subsequently validated experimentally.

### 3.4. Validation of Model-Based Exponential Perfusion Rate

From the multi-objective optimization simulations, a range of perfusion operating scenarios were evaluated by varying the initial fresh medium perfusion rate ($F_{0}$) and the rate at which $F_{0}$ increased over time ($k_{f}$) (Figure 4). Based on the Pareto trade-off between maximizing total secreted FGF2 and minimizing glucose wastage, we selected an exponential perfusion policy defined by $F_{0}=320$ mL h^−1^ and $k_{f}=0.035$ h^−1^. This operating point was chosen because it provided high predicted FGF2 titer while limiting glucose wastage to approximately half of the maximum predicted across the simulated solutions. For experimental validation, cultivation was initiated in batch mode at an initial OD of 0.25. When the culture reached OD 0.5, nisin was added to induce FGF2 expression; this time point was defined as 0 h in all subsequent plots. Perfusion was initiated 4.5 h after induction. During perfusion, the feed flow rate into the bioreactor and the permeate flow rate were maintained as equal to ensure a constant working volume. The applied perfusion profile, an initial $F_{0}$ followed by a gradual exponential increase, is shown in Figure 5d.

The experimental time profiles for biomass (OD_600_), glucose, and lactate are presented in Figure 5a–c together with model predictions (red mean trajectory) and prediction uncertainty (shaded region). The model showed good agreement with experimental measurements across all three state variables, which was further supported by the coefficient of determination (R^2^) and mean absolute error (MAE) values summarized in Table 1. Importantly, lactate remained within the range below that reported to substantially inhibit growth [25,38] throughout the run, while residual glucose was present, indicating that the selected perfusion profile avoided both excessive accumulation of inhibitory by-products and substrate starvation. Under this perfusion policy, the model predicted that, after 8 h of perfusion, the process would reach a secreted FGF2 concentration of ~1136.5 µg L^−1^. When combined with the batch-phase contribution (~300 µg L^−1^), the predicted total FGF2 produced under this condition was ~1436.5 µg L^−1^. The model also predicted a total glucose loss of 7.3 g in the entire perfusion process in this operating condition.

Experimentally, perfusion achieved a final OD_600_ of 49.2 ± 0.35, compared to 6.2 ± 0.2 in batch culture, corresponding to a 7.9-fold increase in biomass (Figure 6a) across one biological replicate. Notably, this OD increase exceeded that previously obtained using repeated semi-continuous exchange cycles in 2 × GM17 medium (Figure 3c). The model predicted a final secreted FGF2 concentration of approximately 1436.5 µg L^−1^ under the corresponding perfusion condition, compared with the experimentally measured value of 2166 ± 161 µg L^−1^. Thus, although the model captured the increase in FGF2 production achieved through perfusion, it underestimated the final experimental concentration by approximately 33.7%. The greater deviation observed for FGF2 compared with biomass, glucose, and lactate likely reflects the simplified representation of recombinant protein production and secretion within the model. In particular, the current model does not explicitly account for potential changes in cellular production capacity, physiological adaptation to prolonged perfusion, or altered protein secretion efficiency at high cell densities. In addition, the cell-retention environment associated with hollow-fiber operation may introduce physiological or transport effects that are not represented by the current kinetic equations. Despite these limitations, the model reproduced the overall process response to perfusion and provided a useful framework for predicting the direction and approximate magnitude of process intensification. Further refinement of the protein production kinetics using additional perfusion datasets could improve the quantitative prediction of FGF2 secretion. Glucose wastage was also consistent with simulation. The final permeates contained an average glucose concentration of 2.25 ± 0.65 g L^−1^ (n = 5). With about 3 L of permeate collected, this corresponds to 6.75 ± 1.95 g of glucose wasted, in close agreement with the simulation-predicted value (7.3 g), with a prediction error of only 7. 5%. Interestingly, intracellular FGF2 increased by only 1.8-fold under perfusion compared to batch (Figure 6b), despite the 7.9-fold increase in biomass. Western blot analysis (Figure 6d) further supported this observation, showing stronger enrichment for secreted FGF2 than for intracellular FGF2 over time. Consistently, SDS–PAGE analysis of purified intracellular and secreted FGF2 at the final time point (Figure 6e) confirmed the higher recovery of secreted and intracellular FGF2 under perfusion compared to batch. Overall, these results provide support for the model-guided exponential perfusion strategy and demonstrate that Pareto front-assisted decision-making provides a rational basis for selecting perfusion parameters that balance recombinant protein titer with nutrient sustainability.

## 4. Discussion

This study demonstrates that perfusion cultivation can overcome limitations associated with the use of *L. lactis* as a recombinant protein production host. By combining hemodialyzer as a cell-retention device for perfusion, and model-guided perfusion feed/bleed strategy, perfusion enabled increases in both biomass concentration and recombinant FGF2 titer while balancing medium wastage. A key finding of this study was the increase in biomass concentration achieved under perfusion operation. Under the exponential perfusion strategy in the fortified medium, *L. lactis* reached an OD_600_ of approximately 49.2 ± 0.35, representing a 7.9-fold increase relative to batch cultivation (6.2 ± 0.2). This biomass level is comparable to or better than those reported in previous *L. lactis* process intensification studies. For example, in situ lactate removal enabled biomass concentrations of approximately 15 g L^−1^ by alleviating lactate accumulation [51,52]. Similarly, the REED^TM^ (Reversed Electro-Enhanced Dialysis) system, which continuously removed lactate during fermentation through electrodialysis, achieved 5- to 10-fold higher OD_600_ than a batch process [53]. Another strategy employed hemin supplementation to promote aerobic respiration in *L. lactis*, reducing lactate formation and enabling biomass concentrations of approximately 5.8 g L^−1^ [14]. Although these approaches employ different mechanisms to enhance growth, they share the common objective of reducing the inhibitory effects of metabolic by-products. Likewise, the perfusion strategy used in the present study continuously supplied fresh nutrients while removing inhibitory metabolites, thereby partially decoupling biomass accumulation from by-product inhibition. The comparable biomass achieved using this approach demonstrates that perfusion can be an effective alternative strategy for the high-cell-density cultivation of *L. lactis*. While the primary objective of this work was recombinant protein production, high-cell-density cultivation is also highly relevant for non-recombinant applications of *L. lactis*, including its use as starter culture and probiotic. The global probiotics market was approximately at least US $70 billion in 2025 [11]. Therefore, the perfusion strategy described here may have broader applicability beyond recombinant protein production wherever higher biomass is desirable. A notable feature of the present system is the use of a commercially available hemodialyzer as the cell-retention device. Unlike dedicated perfusion modules developed specifically for bioprocessing, hemodialysis cartridges are manufactured at large scales for clinical applications, making them widely available and relatively inexpensive. Their standardized manufacturing and lower cost may reduce the economic barrier to implementing perfusion processes, particularly in applications such as food and probiotic production, where process economics are a major consideration. Although further studies are required to evaluate long-term performance, scalability, regulatory suitability, and ethical concern of repurposing products intended for clinical use in food application, these results suggest that hemodialysis cartridges represent a promising use in microbial perfusion cultivation.

Likewise, the increase in biomass translated into increase in recombinant protein production. Secreted FGF2 titers reached approximately 10,416 ± 892 µg L^−1^ in rich medium under semi-continuous perfusion and 2166 ± 161 µg L^−1^ under the continuous perfusion strategy in fortified medium. Importantly, both perfusion approaches improved FGF2 titers more than their corresponding batch controls, demonstrating the effectiveness of perfusion as a strategy for increasing volumetric titer of recombinant protein. These findings indicate that the benefits of perfusion extend beyond higher biomass to supporting higher recombinant protein production as well. The highest secreted FGF2 titer obtained in this study (10,416 ± 892 µg L^−1^) is within the range of recombinant protein titers previously reported for *L. lactis* cultivated in batch processes. In the context of recombinant protein production previously reported for *L. lactis*, Table 2 summarizes published secreted and intracellular recombinant protein titers. The highest secreted FGF2 titer obtained in the present study (approximately 10,416 µg L^−1^) falls within the broader range of recombinant protein titers reported for *L. lactis* and exceeds previously reported secreted FGF2 titers. Nevertheless, direct comparison with published studies should be interpreted cautiously because recombinant protein production is influenced by several factors, including host strain, genetic construct, promoter strength, secretion signal, cultivation conditions, and downstream quantification methods. Therefore, the primary advantage demonstrated in this study is not necessarily achieving a higher absolute titer than previous reports, but rather the improvement of recombinant protein production relative to the corresponding batch process under otherwise comparable conditions.

Interestingly, the increase in protein production was not proportional to the increase in biomass concentration. While cell density increased by nearly 7.9-fold, intracellular FGF2 levels increased by only approximately two-fold, and secreted FGF2 titers did not scale linearly with biomass. This observation suggests that factors other than growth rate influence FGF2 production rate. Although further studies are required to determine the dominant mechanism, these findings highlight an important consideration for process intensification: maximizing biomass concentration alone may not be sufficient to maximize recombinant protein production.

Beyond improving productivity, this study also addresses a critical challenge associated with perfusion processes: balancing process performance with media usage. Increasing perfusion rates generally improves growth and product formation by maintaining favorable cultivation conditions, but it also increases medium consumption and nutrient wastage. As media costs frequently represent a substantial fraction of overall bioprocessing expenses, optimization strategies based solely on maximizing product titer may lead to economically suboptimal operating conditions. To address this challenge, a mechanistic model was integrated with Pareto front to simultaneously consider productivity and substrate utilization. The resulting Pareto front identified a set of non-dominated operating conditions representing different trade-offs between these competing objectives. In particular, a distinct knee region emerged in which substantial improvements in product titer could be achieved with only modest increases in nutrient consumption. Experimental validation of an exponential perfusion strategy selected from this region confirmed that a balanced operating point could be achieved, resulting in improved productivity while limiting unnecessary glucose wastage. These results demonstrate the utility of the model-guided Pareto front as a rational framework for process development and provide an alternative to traditional empirical optimization approaches.

Despite the promising outcomes of this study, several limitations should be acknowledged. First, glucose consumption was used as a proxy for nutrient utilization, whereas other medium components, including amino acids, were not included in the model decision-making. Future studies incorporating comprehensive nutrient balances would provide a more complete assessment of resource efficiency. Second, perfusion experiments were conducted over relatively short cultivation periods, and longer-term operation may reveal additional challenges related to membrane fouling, retention device performance, process sterility, and the genetic stability of the production strain. Third, the kinetic model was developed and validated using data obtained over short cultivation durations and therefore may not fully capture physiological adaptations that occur during prolonged continuous operation. Overall, despite shortcomings, this study demonstrates that perfusion cultivation, coupled with low-cost cell-retention technology and model-guided optimization, can enhance the performance of *L. lactis* as a recombinant protein production platform.

## 5. Conclusions

This study demonstrates the feasibility of repurposing a commercially available hemodialysis cartridge as a low-cost cell-retention device for intensified microbial cultivation, using *L. lactis* for recombinant FGF2 production as a case study. Compared with batch operation, the cartridge-enabled perfusion setup increased biomass accumulation and improved secreted FGF2 yield under the conditions evaluated, indicating that such an approach can enhance volumetric titer. In addition to experimentally evaluating perfusion operation, this work integrates a model-based simulation framework with multi-objective optimization to support rational process decision-making. The Pareto analysis highlighted that perfusion intensification involves a trade-off between increasing secreted FGF2 production and minimizing glucose wastage. Selection of a knee-point perfusion strategy provided a practical compromise and enabled experimental validation of a balanced operating condition. This modeling and decision framework may be applicable to other perfusion-based microbial processes where competing objectives must be considered. Overall, the results suggest that hemodialysis cartridges can serve as an accessible option for cell retention in laboratory-scale microbial perfusion studies, and that model-guided decision-making will assist in future intensified microbial biomanufacturing.

## Figures and Tables

**Figure 1:**
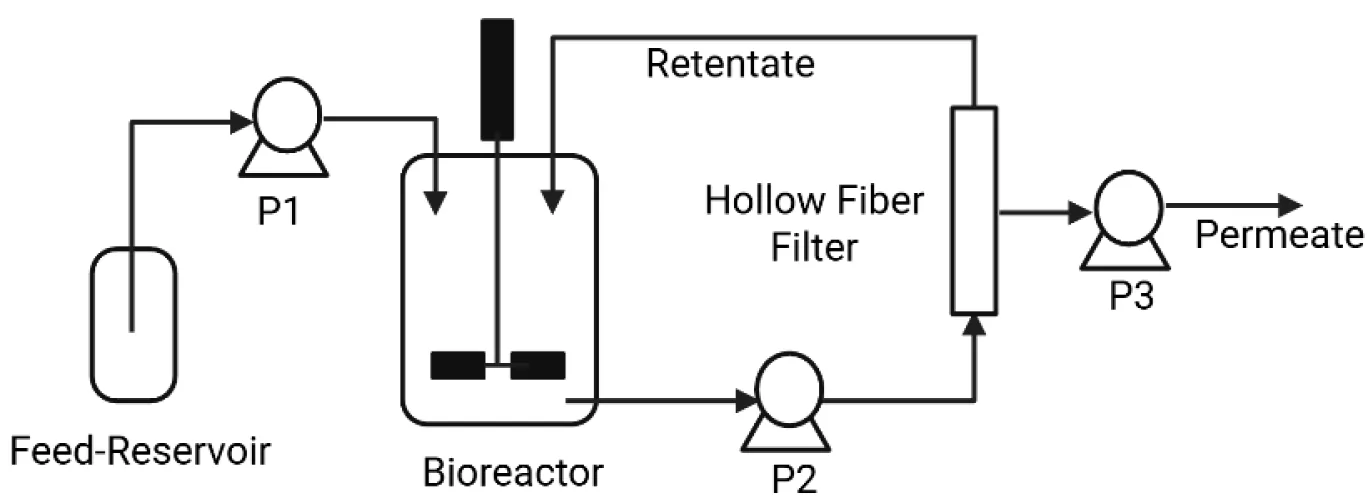
Schematic of the perfusion process.

**Figure 2:**
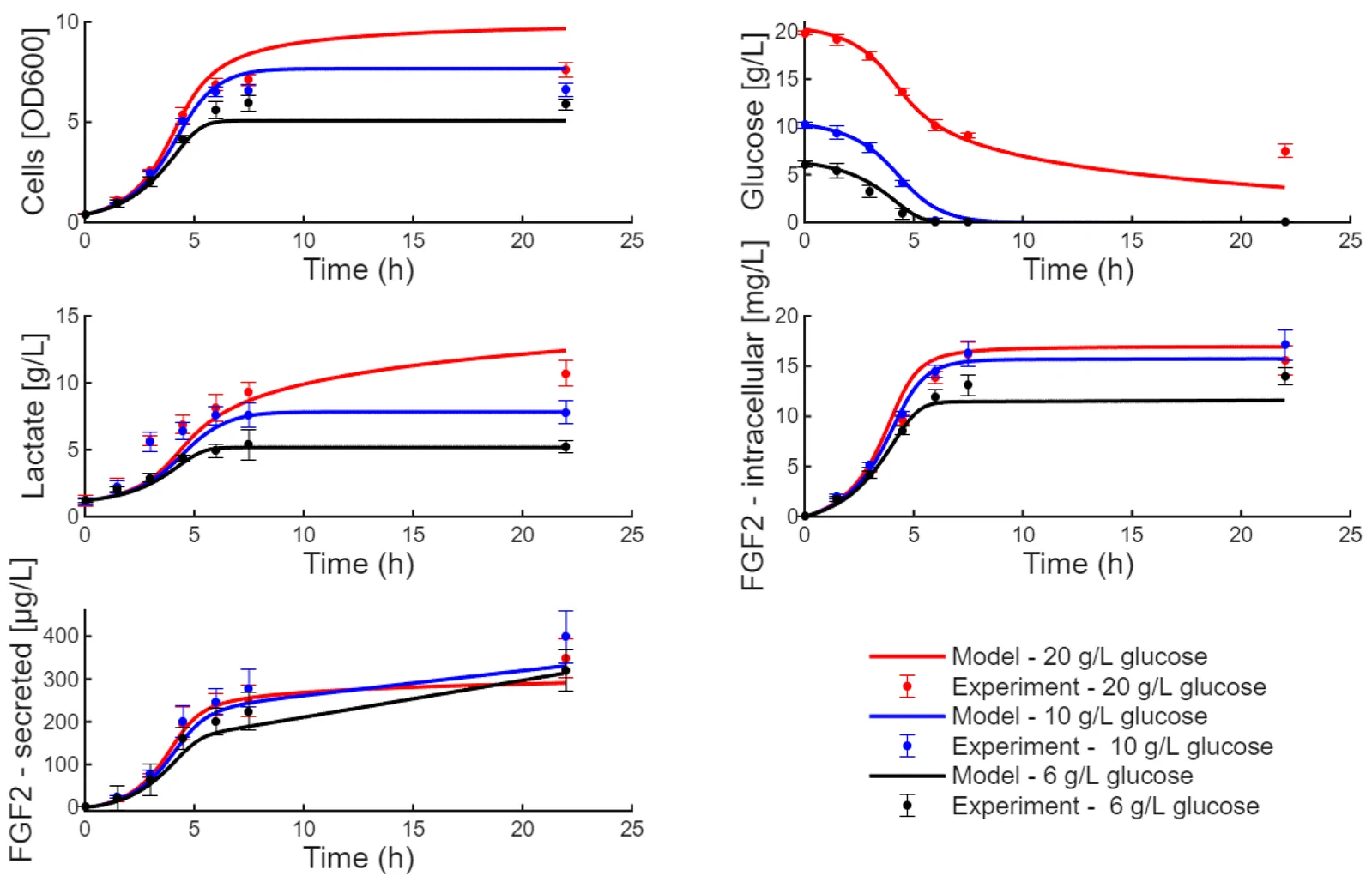
Experimental data from a batch fermentation for cells (OD_600_), glucose, lactate, intracellular FGF2, and secreted FGF2 obtained at three different initial glucose concentrations (6, 10, and 20 g L^−1^) were compared with the corresponding model predictions. The solid lines represent the average prediction of 20 simulations, Nisin induction was performed at 0 h. Data from all three glucose conditions were used for model parameter estimation.

**Figure 3:**
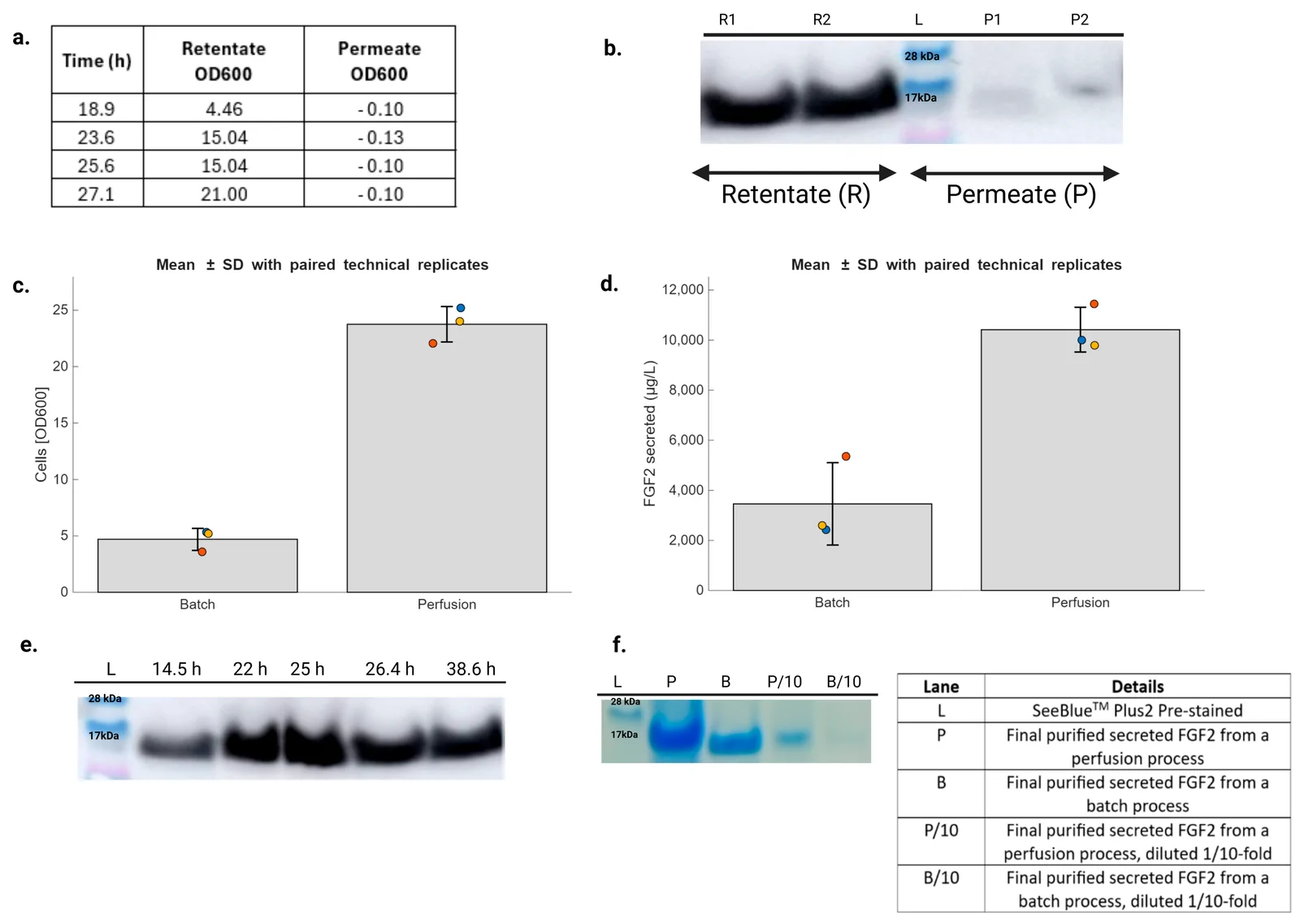
(**a**) OD600 value in the retentate and permeate at different time points. (**b**) Western blot image of secreted FGF2 in retentate and permeates fraction. (**c**) Comparison of final OD in a batch and perfusion process (n = 3, biological replicate). (**d**) Comparison of final secreted FGF2 concentration in a batch and perfusion process (n = 3, biological replicate). (**e**) Western blot image of secreted FGF2 at different time points in one of the perfusions run. (**f**) Purified secreted FGF2 from a batch and perfusion process visualized in SDS image. L: molecular weight ladder (SeeBlue^TM^ Plus2). P: final purified FGF2 from a perfusion process. B: final purified FGF2 from a batch process. P/10: final purified FGF2 from a perfusion process loaded after 10-fold dilution. B/10: final purified FGF2 from a batch process loaded after 10-fold dilution.

**Figure 4:**
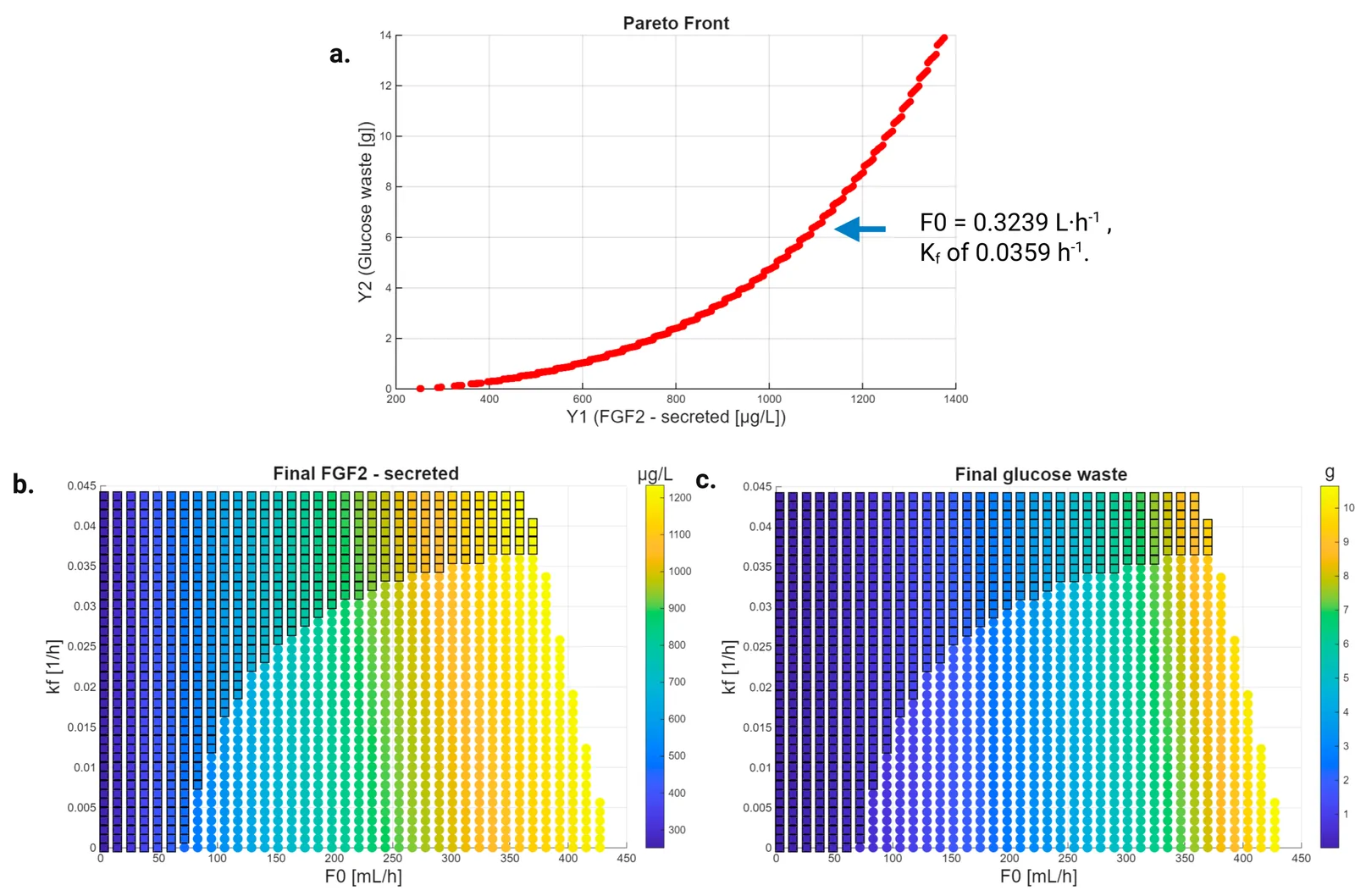
(**a**) Pareto front obtained from multi-objective optimization of perfusion operation by simulating multiple combinations of the initial perfusion rate, F_0_ (mL h^−1^), and the perfusion ramp parameter, $K_{f}$ (h^−1^), which defines the rate of increase in perfusion flow over time. Each point represents a unique (F_0_, $K_{f}$) perfusion strategy evaluated over an 8 h cultivation. Two competing objectives were considered: maximization of secreted FGF2 titer, and minimization of glucose wastage, quantified as residual glucose in the harvest stream. The Pareto front represents the set of non-dominated solutions defining the best achievable trade-offs between these objectives. The knee point identifies a practical compromise solution where further improvement in one objective requires a disproportionately large loss in the other. The knee-point strategy was selected for experimental validation. (**b**) Heatmap of simulated total secreted FGF2 (µg L^−1^) across the (F_0_, k_f_) design space. Simulations were performed for 8 h using initial conditions: OD_600_ = 5, glucose = 2 g L^−1^, lactate = 5 g L^−1^, and working volume = 500 mL (0.5 L). Pareto-optimal strategies are indicated by square markers, whereas non-Pareto solutions are shown as circular markers. Regions without markers correspond to infeasible strategies that violated the perfusion feed volume constraint (total perfused volume > 3.5 L). (**c**) Heatmap of simulated total glucose waste (g) across the same (F_0_, k_f_) design space, with identical initial conditions and feasibility constraint. Marker definitions are the same as in panel.

**Figure 5:**
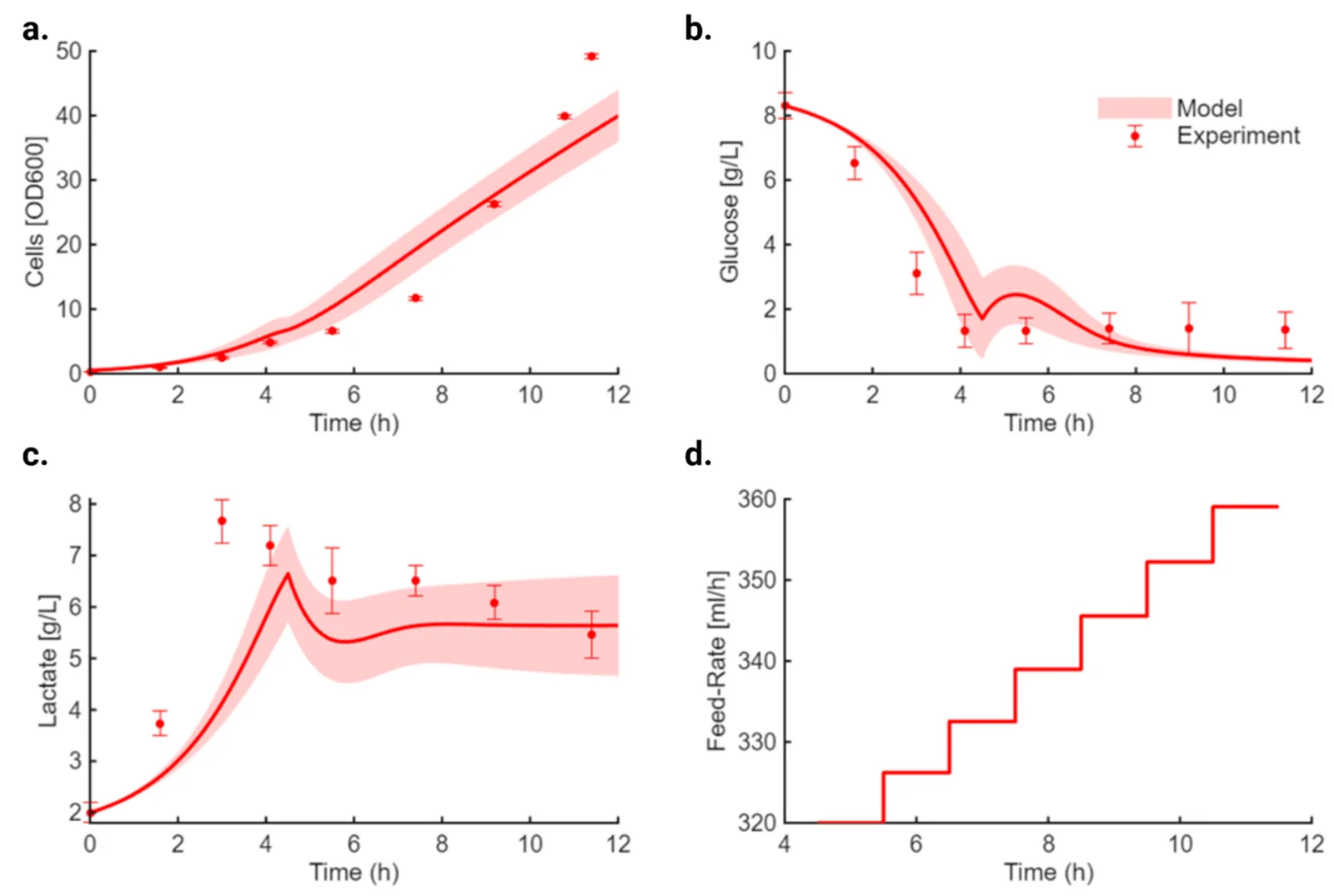
(**a**–**c**) Experimental data for cells (OD_600_), glucose and lactate plotted against model predictions. The solid line represents the average prediction of 20 simulations, with the shaded area denoting the prediction’s lower and upper bounds. Nisin induction was performed at 0 h. Perfusion process started at 4.5 h. (**d**) Profile of perfusion flow rate ($F_{0}$) in mL h^−1^ at different time points.

**Figure 6:**
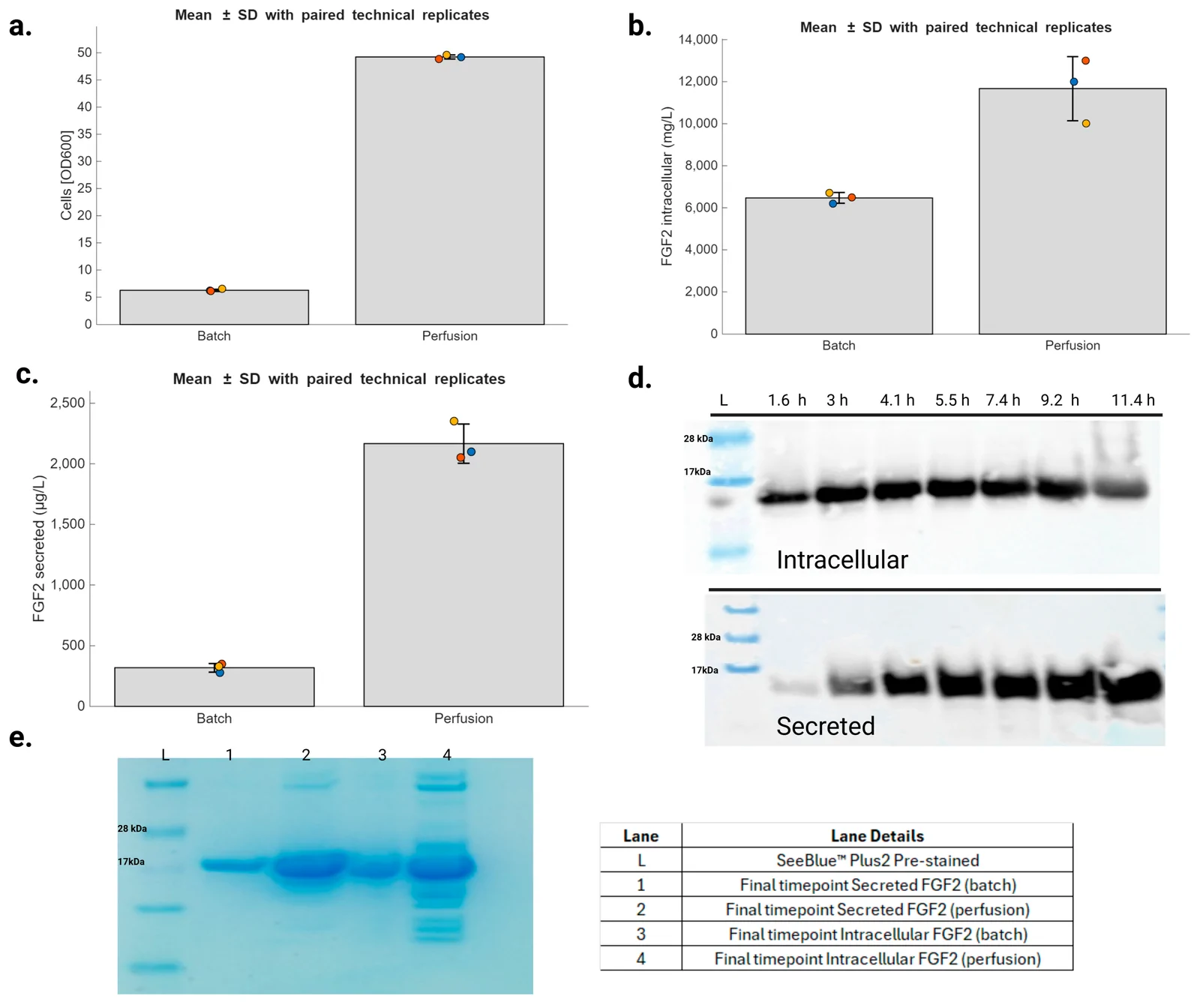
(**a**) Comparison of final OD in a batch and perfusion process when an exponential perfusion rate was used (n = 1). (**b**) Comparison of final intracellular FGF2 in a batch and perfusion process when an exponential perfusion rate was used (n = 1). (**c**) Comparison of final secreted FGF2 in a batch and perfusion process when an exponential perfusion rate was used (n = 1). (**d**) Western blot image of intracellular FGF2 (top) and secreted FGF2 (bottom) at different time points in a perfusion process. (**e**) Purified finaltime pointt secreted and intracellular FGF2 from a batch and perfusion process visualized in SDS image. L: molecular weight ladder (SeeBlue^TM^ Plus2). 1: final time point secreted purified FGF2 from a batch process. 2: final time point secreted purified FGF2 from a perfusion process. 3: final time point purified intracellular FGF2 from a batch process. 4: final time point purified intracellular FGF2 from a perfusion process.

**Table 1:** R^2^ and MAE values of the model prediction for each process variables.

Variables	R^2^ Value	MAE
Cells	0.94	3.38
Glucose	0.85	1.0
Lactate	0.67	1.74

**Table 2:** The titer of different recombinant proteins produced by L. lactis in the literature.

Protein	Secreted Recombinant Protein Titer	Intracellular Recombinant Protein Titer	References
Nuc	20,000 µg L^−1^	3000 µg L^−1^	[54]
L7/L12	3000 µg L^−1^	500 µg L^−1^	[55]
E7 (rE7)	35,000 µg L^−1^	-	[56]
FGF2	1970 µg L^−1^	-	[15]
FGF2	396 µg L^−1^	16,000 µg L^−1^	[57]

## Data Availability

The original contributions presented in this study are included in the article/Supplementary Materials. Further inquiries can be directed to the corresponding author.

## Supplementary Materials

The following supporting information can be downloaded at https://www.mdpi.com/article/10.3390/microorganisms14102191/s1. Table S1: Metabolite composition of different media including fortified spent media used in this study. Metabolite concentrations were quantified using a BioProfile 100 Plus analyzer, while total protein and total free amino acids were determined by Bradford and ninhydrin assays, respectively. Table S2: The model parameters along with their description, unit, values, initial guesses used for the optimization, values after parameter estimation, and respective lower and upper bound constraints during the parameterization process.
