# Supplementary material for: Bioprocess Intensification Using Perfusion Culture for Recombinant Protein Production from Lactococcus lactis
Source: Microorganisms. 2026 Oct 1;14(10):2191. doi: 10.3390/microorganisms14102191 (PMC13707899; doi:10.3390/microorganisms14102191)
Supplement: Supplementary file 1 [file microorganisms-14-02191-s001.zip › microorganisms-4545923-supplementary.pdf]

Supplementary Material

Table S1: Metabolite composition of different media including fortified spent media used in this study. Metabolite concentrations were quantified using a BioProfile 100 Plus analyzer, while total protein and total free amino acids were determined by Bradford and ninhydrin assays, respectively.

**Components (units)**	**Spent media**	**Fortified Spent Media**	**2GM17 + 2% Glucose**
pH	6.78 ± 0.12	7.3 ± 0.32	7.23
Glutamine (mM)	1.78 ± 0.21	2.9 ± 0.29	0
Glutamate (mM)	0.49 ± 0.12	3.4 ± 0.34	4.35
Glucose (g/L)	1.95 ± 0.17	1.8 ± 0.22	22.3
Lactate (g/L)	1.53 ± 0.13	1.7 ± 0.19	2.95
NH4+(mM)	0.92 ± 0.14	2.7 ± 0.35	3.08
Na+(mM)	143.0 ± 6.73	147.6 ± 7.2	183.3
K+(mM)	4.03 ± 0.19	19.5 ± 6.1	24.4
Total protein (mg/L) (Bradford assay)	Not detected	70.5 ± 11.2	383 ± 26
Total free amino acids (g glycine equivalent/L) (Ninhydrin assay)	0.78 ± 0.23	8.42 ± 0.77	5.05 ± 0.58

Parameters Value

Table S2: The model parameters along with their description, unit, values, initial guesses used for the optimization, values after parameter estimation, and respective lower and upper bound constraints during the parameterization process.

S.N.	Parameter	Description (Unit)	Optimized Value	Initial Guess	lower bound	upper bound
1	Y_{XS}	OD Yield coefficient from glucose (OD g^{-1})	0.7641 ± 0.0662	1.6	0.5	2.2
2	$L_{i,\mu}$	lactic acid inhibition in growth rate	10.1 ± 0.7406	8	3	18
3	$n1$	hill coefficient	5	3	2	7
4	qS_{max}	Maximum Specific glucose consumption rate (g OD^{-1} h^{-1})	0.855 ± 0.0488	0.95	0.45	3
5	K_s	Saturation constant (g L^{-1})	1.0824 ± 0.0562	2.05	1	4
6	$L_{i,qS}$	Inhibition constant (lactate on glucose uptake) (g L^{-1})	6.5 ± 0.21	7	3	14
7	$n2$	hill coefficient	5	3	2	7
8	qL_{max}	Maximum Specific lactate production rate (g OD^{-1} h^{-1})	1.625 ± 0.0783	0.635	0.3	1.5
9	K_{S_L}	Saturation constant (g L^{-1})	1.527 ± 0.3999	1.05	0.5	2
10	α_i	Specific FGF production rate – growth associated (µg OD^{-1} h^{-1})	1837.5 ± 19.17	1800	400	25000
11	$L_{i,qP_{intra}}$	Inhibition constant (lactate on intracellular FGF production) (g L^{-1})	9 ± 1.9212	6	3	14
12	$n3$	hill coefficient	5	3	2	7
13	α_s	Specific FGF secretion rate – growth associated (µg OD^{-1} h^{-1})	16.76 ± 2.5985	10	8	30
14	β_s	Specific FGF secretion rate – non growth associated (µg OD^{-1} h^{-1})	2.1030 ± 0.1682	2	0.05	5
15	K_{FGF2_i}	Saturation Constant (μg^{-1} L^{-1})	100.580 ± 0.857	100	1	250
16	$L_{i,qFGF2_{sec}}$	Inhibition constant (lactate on secreted FGF production) (g L^{-1})	10 ± 0.82	7	3	14
